# A Priori Reasoning as to Pathogeny

**Published:** 1917-05

**Authors:** 


### A Priori Reasoning as to Pathogeny.

With the example of the late President Lincoln, we offer no apology for repeating this story: A hired man, discharged for coming to work late, went to his room and took his alarm clock to pieces. His comment was as follows: "No wonder it didn't go off, the engineer is dead." He was an ignorant man of course, but how about the physicians who reported cases of yellow fever derived from fomites, as from a lock of hair years after death; how about the classification of malaries; how about the demonstration that the germ of syphilis must be ultra-microscopic because the virus passed through a porcelane filter, whereas we now know that the real germ is about the largest of any with which we have to deal? And, by the way, how about the ridicule of the non-committal word **germ** and the tendency to supplant it by the word **basterium?** Count the specific diseases of known micro-organismic etiology and, unless you have given the matter special attention, you will be surprised at the number of infections which are due to **germs** that are not bacteria. A great many elaborate theories of pathogeny, have been ruthlessly shattered, though as fully accepted, as the specificity of rabies and the present theory of immunization.

